# mmSTORM: Multimodal localization based super-resolution microscopy

**DOI:** 10.1038/s41598-018-37341-9

**Published:** 2019-01-28

**Authors:** Tamás Gajdos, Zsófia Cserteg, Szilárd Szikora, Tibor Novák, Bálint Barna H. Kovács, Gábor Szabó, József Mihály, Miklós Erdélyi

**Affiliations:** 1 Department of Optics and Quantum Electronics, University of Szeged, 6720 Szeged, Dóm tér 9 Hungary; 2 BRC Institute of Genetics, Biological Research Centre HAS, Szeged, Hungary; 3 MTA-SZTE Research Group on Photoacoustic Spectroscopy, Szeged, Hungary

## Abstract

Super-resolution localization microscopy provides a powerful tool to study biochemical mechanisms at single molecule level. Although the lateral position of the fluorescent dye molecules can be determined routinely with high precision, measurement of other modalities such as 3D and multicolor without the degradation of the original super-resolved image is still in the focus. In this paper a dual-objective multimodal single molecule localization microscopy (SMLM) technique has been developed, optimized and tested. The proposed optical arrangement can be implemented onto a conventional inverted microscope without serious system modification. The performance of the method was tested using fluorescence beads, F-actin filaments and sarcomere structures. It was shown that the proposed imaging method does not degrade the image quality of the original SMLM 2D image but could provide information on the axial position or emission spectra of the dye molecules.

## Introduction

Single molecule localization based super-resolution microscopy (SMLM) methods such as PALM^1,2^, FPALM^3^, STORM^4^, dSTORM^5^ and GSDIM^6^ provide the highest spatial resolution images captured via optical microscopy techniques. In these methods temporally and spatially isolated fluorescent molecules are imaged using traditional wide-field microscope systems. The images of these point-like single emitters are fitted by the theoretical point spread function (PSF) the center of which defines the lateral position of a molecule. A strictly controlled photoswitching process is required to keep the number of active fluorescent molecules at an optimum value during the data acquisition^7^. In case of a high labeling density, the spatially overlapping PSFs preclude the precise localization of independent molecules, while at a low density the increased acquisition time limits the final image quality^8^. SMLM is a photon number limited technique. Single fluorescent dyes can typically emit a few thousand photons during a single ON stage, which determines the localization precision and consequently the final image quality^9,10^. However, the emitted photons also carry information on the axial position and the local environment of the dye molecules. The question is how to read out this extra information and reveal additional physical and chemical properties of the sample at single molecule level?

Different PSF engineering methods have been proposed to determine the axial position of single fluorescent molecules. Astigmatic^11–13^, biplane^14,15^, double helix^16,17^, interferometric dual objective^18^ and Airy-beam^19^ approaches have been proposed to code the axial position of a single molecule into the shape of the PSF. The stronger the dependency, the higher the axial localization precision. However, a serious distortion of the PSF can reduce both the axial range of the localization and the lateral resolution. PSF engineering methods are quasi-3D methods, since the mapped axial range is still limited by the depth-of-field (DOF), which is typically at around 1 micron. Real 3D SMLM imaging requires either optical or mechanical sectioning^20^ and is a time-consuming process.

In addition to the 3D spatial position of the fluorescent molecules, the emitted photons also provide information on the orientation^21–30^, the rotational/translational mobility and diffusion^31^ and the local environment of the molecules. These properties can be determined via the measurement of physical parameters such as polarization (anisotropy)^32–34^ and fluorescence emission spectra^35–37^. However, modification of the PSF typically affects the overall final image quality and requires a trade-off between different modalities due to the limited photon number.

The 4Pi method^38^ is based on a dual-objective configuration generating an interference pattern via counter-propagating beams inside the sample. Using the 4Pi type C configuration, the emitted light is collected by both objectives, which results in high detection efficiency. In the double-pass confocal transmission system interference is generated between the direct and the back reflected beams^39^. Several dual-objective configurations have been proposed for SML microscopy, when the two images are captured by either a single^40–45^ or multi camera^18,46^ setup. Single camera techniques capture images simultaneously under the very same acquisition conditions that typically make post processing data analysis easier. It is worth noting that SMLM image stacks are acquired at reduced frame size (typically smaller than half of the full frame size), therefore single camera approaches reduce the final super-resolved ROI. Multi camera configurations^18,46^ can use the entire frame size, however, they require precise synchronization of the cameras and additional post processing steps, especially when different detectors are applied^46^. Dual objective configurations mostly apply two optically balanced pathways to eliminate aberrations. Such configurations require the application of identical optical components in the two arms and stable optical stages. For example, the interferometric fluorescent 3D super-resolution approach generates and captures three interference patterns under different retardation conditions using identical CCD cameras, and determines the axial position of fluorescent molecules with sub 20 nm resolution^18^.

In this paper we report the development and application of an optical setup for multimodal SMLM that can be potentially used for 3D, polarization and spectral sensitive measurements. The proposed system has several advantages over other configurations. It does not affect the primary SMLM imaging process, and yields a normal 2D super-resolved primary image. However, a secondary image is also generated independently by photons emitted in the direction opposite to the primary objective. The primary (P) and secondary (S) images are mirror images and are captured simultaneously by a single detector array. The arrangement requires no system modification on the detector port and keeps the original optical axis. This makes switching between the normal and multimodal modes easy and straightforward, and simplifies system alignment. The paper is arranged as follows: First, the multimodal optical system is described and a comparative evaluation of S and P PSFs is given. In the next section different modalities (3D astigmatic, 3D biplane, multicolor) are tested and evaluated. Finally, the discussion and conclusions are presented.

## Optical Setup Evaluation Based on Simulated and Measured PSF

**Figure 1:**
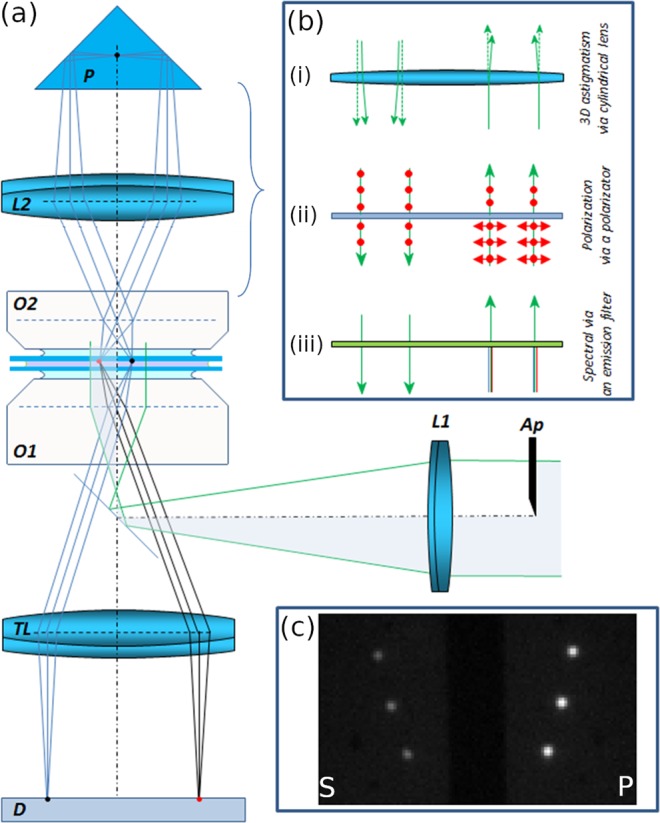
Experimental setup of mmSTORM configuration. (**a**) Different modification options of the secondary image are possible via astigmatic 3D (i), polarization (ii) or spectral (iii) filters inserted between the objective and the prism. (**b**) Captured image frame with primary (P) and secondary (S) images of three beads (**c**).

The proposed experimental setup was built on a standard inverted fluorescent microscope (Fig. 1(a)). The collimated excitation beam (green lines) was focused into the back focal plane of the primary microscope objective (O1). In the EPI illumination mode half of the beam could be blocked by an aperture (Ap) placed in front of the focusing lens (L1), therefore only half of the original field of view (FOV) was excited. Although the excited fluorescent molecules emit photons in the full 4Pi solid angle, the traditional image is generated by photons captured by the microscope objective O1. Photons leaving the sample in the opposite direction typically do not contribute to the imaging process. Consequently, more than 50% of the emitted photons are lost even when extremely high numerical aperture (NA) objectives are used. The final image of a single fluorescent molecule (red dot) is formed on the detector (D) by means of an additional tube lens (TL), and can be described as the primary point spread function of the optical system. The black solid lines depict the image formation in the primary path. In the proposed dual objective configuration a secondary microscope objective (O2) was aligned opposite the primary one to collect photons emitted upwards. The numerical aperture of the applied objectives were 1.49 (O1) and 1.4 (O2), wherefore the emitted photons could be collected with high efficiency. When a secondary tube lens (L2) and a Porro prism were used, the secondary objective formed a mirror image in the other half of the FOV (black dot). In the secondary arm a spectral filter can be inserted to attenuate the excitation beam, eliminating the excitation of the sample via the back reflected beam. Alternatively, TIRF or HILO excitation can be applied to drastically reduce the throughput of the excitation beam (NA_secondary_ < NA_primary_). During the measurement we used Hilo mode for the reduction of out of focus fluorescence and also to eliminate the excitation effect of the back reflected excitation beam. The secondary image was also generated via the primary objective, and the detector captured the P and S images simultaneously. The primary and secondary images of three fluorescent beads can be seen in Fig. 1(c). Although all the optical components inserted into the secondary path were AR coated (see Section Optical Setup of Supplement), the intensity of the secondary image was typically around 50% of the primary one. The most significant source of light loss was the secondary microscope objective (T_647nm_ = 81%, T_561nm_ = 84%). For this reason the primary image was applied to determine the lateral position of the molecules, while the secondary image was used to evaluate the selected modality.

The secondary arm is an open-path system, where the emitted light can be manipulated relatively easily by inserting different optical components. Three modalities are depicted as examples in Fig. 1(b). Astigmatism, which can be introduced by a cylindrical lens (Fig. 1(b,i)), encodes the axial position of the dye molecules into an elliptical shape of the PSF and makes localization precision anisotropic, which is typically mentioned as the main drawback of this approach. Our method does not degrade lateral super-resolution, and nor does it introduce lateral anisotropy. Furthermore, it keeps the primary image unaffected, since the axial position is only determined by the secondary image. It is worth noting that biplane 3D axial super-resolution can also be implemented by introducing a slight defocus in the secondary arm. The emitted, partially polarized fluorescent light (Fig. 1(b,ii)) can also be filtered by a polarizer, selecting one particular polarization component (red dots in Fig. 1(b,ii)). In this case the captured intensities of the primary and secondary images are proportional to the total intensity and the selected linearly polarized intensity component, respectively. After a calibration process the polarization degree ($P=({I}_{\parallel }-{I}_{\perp })/({I}_{\parallel }+{I}_{\perp })$) or fluorescence anisotropy ($r=({I}_{\parallel }-{I}_{\perp })/({I}_{\parallel }+2{I}_{\perp })$)^30,34^ can be determined. The emitted fluorescence light can also be filtered spectrally (Fig. 1(b,iii)) in the secondary arm, so samples labeled with two different dyes can be imaged simultaneously. The real implementation of the secondary path is depicted in Fig. S1 of Supplement. During the experiments the sample was sandwiched between two coverslips (see Fig. S2 of Supplement), and the thickness of the buffer (PBS or switching) layer was typically around 25 microns. The secondary and primary objectives were corrected for standard (0.17 mm) and variable (0.13 mm–0.19 mm) cover slide thicknesses, respectively. To minimize the optical aberrations introduced by the objectives, the sample was fixed to the surface of the upper coverslip and the axial distance was measured from this reference surface throughout this paper. The microscope objective in the secondary arm (O2) was a PLAN objective to minimize image curvature.

**Figure 2:**
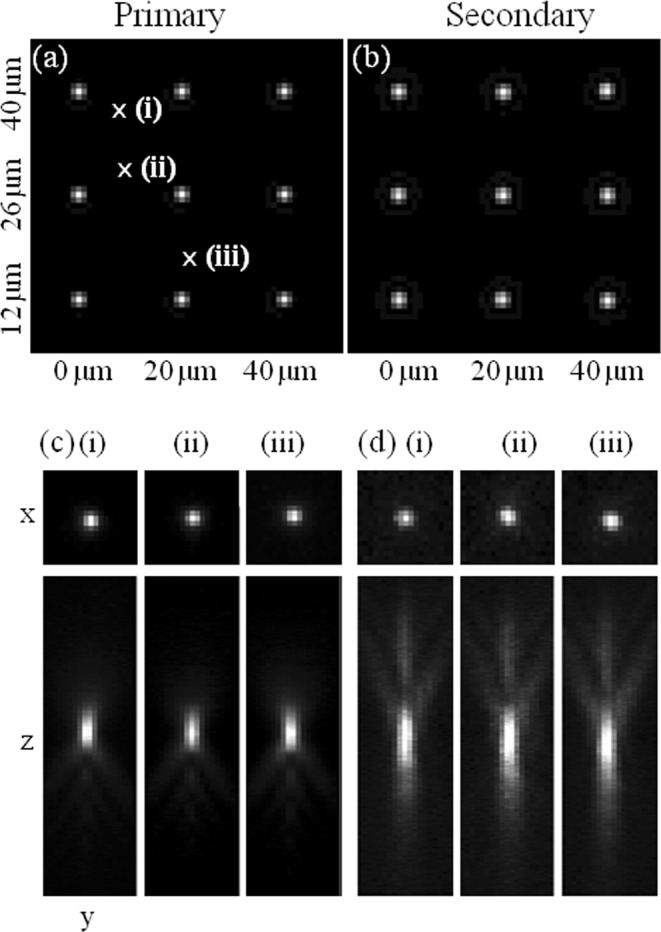
Simulated primary and secondary images of fluorescent molecules situated at lateral x (0 μm, 20 μm, 40 μm) and y (12 μm, 26 μm, 40 μm) off axis coordinates are depicted (**a**,**b**) respectively. Experimentally measured lateral (x, y) and axial (z, y) intensity distributions of the primary (**c**) and secondary (**d**) images of three fluorescent beads in the positions marked by x (**a**). The pixel size is 160 nm.

Since the secondary path is a complex system that contains several optical components the misalignment of which can introduce significant optical aberrations, the complete system was modeled (see the OSLO model in Fig. S3 and more details on simulations in the Computer simulation of the optical setup section of the Supplement), and the calculated primary and secondary PSFs were evaluated by OSLO (Optics Software for Layout and Optimization)^47^. Figure 2 depicts the comparative simulated (a and b) and measured (c and d) PSFs. The total field of view (FOV) limited by the overall magnification (x100) and the chip size of the detector (8.2 × 8.2 mm^2^) was 80 × 80 μm^2^ on the sample. Due to the symmetry of the system only one quarter of the FOV is shown. Primary and secondary images of simulated fluorescent molecules situated at x (0 μm, 20 μm, 40 μm) and y (12 μm, 26 μm, 40 μm) coordinates are depicted in Fig. 2(a,b), respectively. Image formation close to the y = 0 axis was not possible because of the polished edge of the applied Porro prism. In the captured images a black strip between the primary and the secondary images represents this effect (Fig. 1(c)). A quantitative evaluation of the primary and secondary images was given based on fitting 2D Gaussian distribution to all the simulated PSFs. Neither the primary, nor the secondary images show spatial dependence. The standard deviation of the fitted FWHM values was typically found to be below 2%. However, the Gaussians distribution fitted to the secondary images were found to be approximately 15% broader than the primary ones, presumably because of optical aberrations introduced in the secondary path. Ellipticity, defined as the ratio of the standard deviations of the fitted Gaussian curves in x and y directions (ε = σx/σy − 1 if σx > σy; and ε = −σy/σx + 1 otherwise) has also been found to be independent of the lateral position of the molecule (error <1%). The ellipticity calculated from measured 100 nm fluorescent beads showed no signs of lateral dependence. The deviation was caused by background noise and fitting. The experimentally captured 3D PSF (Fig. 2(c,d)) show a very good agreement with the simulation data (see Fig. S4 of the Supplement). The P and S images of three fluorescent beads in the positions marked by x reveal a slight image degradation in the S channel. Although the images show normalized intensities, the Strehl ratio of S images were typically around 50% of the P ones. The calculated 3D PSFs as functions of position z also prove the applicability of the proposed setup for SMLM.

## Experimental Validation of 3D and Multicolor Modalities in SMLM

### Comparison of primary and secondary dSTORM images

**Figure 3:**
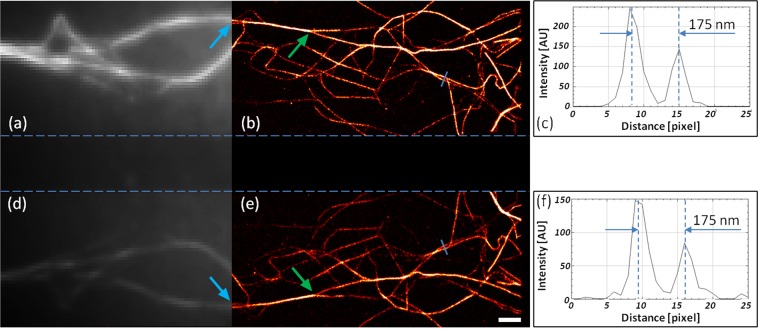
Primary (**a**,**b**) and secondary (**d**,**e**) traditional fluorescence (**a**,**d**) and dSTORM super-resolved (**b**,**e**) images of F-actin filaments fixed to the coverslip. Intensity cross-sections through the marked blue lines in the primary (**c**) and secondary (**f**) images show minimal image degradation. The scale bar is 1 micron.

Image stacks on F-actin filaments labeled by phalloidin-Alexa647 [ThermoFisher A22287] were captured for 2D dSTORM imaging. After the cleaning (piranha solution) and drying steps the coverslip was covered with poly-L-lysine. Labeled and washed F-actin filaments in their original buffer were dropped onto the prepared surface and incubated for 5 minutes. After additional washing steps the buffer was changed to dSTORM switching buffer^5^, and the sample was covered with a second cleaned coverslip. The prepared sample was mounted onto the microscope in the very same way as discussed in the previous section. Image stacks of 10,000 frames were captured and evaluated by rainSTORM code written in Matlab^48^. Figure 3(a,d) show the primary and secondary diffraction limited fluorescence images. The super-resolved primary and secondary dSTORM images are depicted in Fig. 3(b,e), respectively. The central part (the region between the primary and secondary images) is blank because of the polished edge of the applied prism. The improved resolution can be clearly seen in both cases. For example, the double actin filament marked with blue arrow can be seen clearly separated both on the P and S images, while they appear as single lines on the diffraction limited images. One can also realize that due to the reduced photon number in the S arm, the number of accepted localizations is lower in the S image than in the P one. However, the relative intensities of the individual fibrils remained the same. The green arrows mark a branch region where the intensity ratio of individual branches kept the same value. Due to the reduced photon number and the minimal image distortion introduced by the secondary arm, the quality of the S image is still high enough to resolve structures below the diffraction limit. Figure 3(c,f) depict the cross-sections of primary and secondary images along the blue lines. Despite the reduced number of localizations, both images provide the same separation of the selected fibrils and also keep their intensity ratio.

A statistical comparative evaluation of primary and secondary localizations can be seen in Fig. S5 (see section Comparative evaluation of the primary and secondary localizations of Supplement). The principal reason of the reduced number of localizations in the S image is the loss of intensity (photon number) in the secondary arm. The lower the intensity the higher the degradation of the captured PSFs. Localization software (rainSTORM) judges the quality of individual spots and rejects low quality (faint, elliptic, broad etc.) ones. Owing to this selection, the secondary image contains fewer localizations, when the same threshold values are used. A pair finding algorithm then pairs the localizations from the primary and from the secondary image to calculate the desired modality. The code follows a two-step process. First a rough estimation is made based on the evaluation of all possible localizations. At this step we assume that ideally (perfect optical alignment and aberration free imaging) the sum and difference of the appropriate coordinates of the pairs are constant (and are equal to the frame size) and zero, respectively. During the second step the algorithm searches the close vicinity of the ROI determined in the previous step and judges the localizations found (for more details see the Pair finding algorithm section of the Supplement).

### Astigmatic 3D dSTORM modality

**Figure 4:**
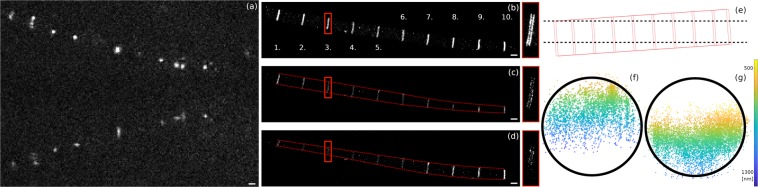
Implementation of astigmatic 3D imaging in mmSTORM. The primary and secondary PSFs of Alexa Fluor dye molecules excited at 647 nm show circular and astigmatic shapes in a single image frame (**a**). The double disc structure of H-zone in sarcomeres has different visibility and length in the unfiltered (**b**) and in the STORM images reconstructed from two selected planes (**c**,**d**). The two optical sections (**c**,**d**) separated by 230 nm are marked in the schematic view of the tilted structure (**e**). Circles with a radius of 0.8 μm were fit to the cross-sections of zones 3 and 8 (**f**,**g**). The scale bar is 1 micron.

The astigmatic 3D imaging modality of the proposed optical setup was experimentally demonstrated by means of imaging an F-actin pointed-end-capping protein, tropomodulin (Tmod) in sarcomers^49^, and Alexa 647 labelled microtubules in Schneider 2 cells. The detailed preparation protocols of the samples are given in the Tissue preparation and immunostaining section of the Supplement. The results of 3D astigmatic imaging of microtubules are also presented in the Supplement (S9). In this section we focus on the 3D imaging of sarcomere sample. During the measurements 30,000 frames were captured and evaluated. Figure 4(a) shows a typical single frame from the acquired image stack when an extra cylindrical lens with a focal length of 4000 mm (Comar 4000 Y 25) was inserted into the secondary arm (see optical arrangement in Fig. 1(b,i)). The focal length and insertion point of the cylindrical lens were optimized using a matrix optics model. Figure S6 depicts the schematic optical arrangement and Fig. S7 the calculated separation of foci as a function of the location of the cylindrical lens. (Calculation is detailed in the Optimization of the introduced astigmatism based on paraxial matrix optics method and experimental calibration section of the Supplement). The experimental calibration curves as a function of the insertion point of the cylindrical lens in the secondary arm can be seen in Fig. S8. The orientation of the cylindrical lens was set so that the minor and major axes of the elliptical PSF be parallel to the x and y coordinates, respectively. While the spots in the upper section (P image) show cylindrical symmetry, the PSFs in the lower section (S image) have the well-known astigmatic shape. Fluorescence beads fixed to the coverslip were used to determine the z position as a function of ellipticity of the measured PSFs. Figure 4(b) shows the normal, unfiltered 2D dSTORM image, while Fig. 4(c,d) are two ~46 nm thin sections of the reconstructed volumetric image at different z positions (915 nm and 685 nm). The dashed red lines mark the contour of the sarcomere. In Fig. 4(b) the width of the H-zone remains practically the same all the way through the myofibril, while the two z-section images (Fig. 4(c,d)) reveal the cylindrical shape distribution of the labeled sample. The magnified images of the third zone (framed with red) clearly show the double line structure and their change in length as a function of depth. It is worth noting here that the double line separated by approximately 125 nm in a single zone cannot be resolved in traditional diffraction limited microscopy. The depth (axial) distribution of localizations belonging to a single disk was also visualized (Fig. 4(f,g)). Since the depth of field (DOF) determining the axial region for 3D imaging is around 600 nm and smaller than the typical width of the sarcomere (1.6 μm) only a section of the disc can be imaged. Figure 4(f,g) show the cross-section of disks number 3 and 8. One can realize the axial shift between the two discs caused by the tilted sample (Fig. 4(e)). The measured radius of the disk was found to be 0.8 μm and is in good agreement with previous measurements^50^.

The STORM images were generated using localizations with a precision better than 50 nm. The mean and median values of these accepted localizations were 18.21 nm and 15.6 nm, respectively. The axial localization precision was calibrated using fluorescence beads, and given as the standard deviation of the calculated Z positions. Based on the evaluation of the measured data of 20 beads, the axial localization precision and the axial range were found to be 58 nm and 700 nm, respectively.

### Biplane 3D dSTORM modality

**Figure 5:**
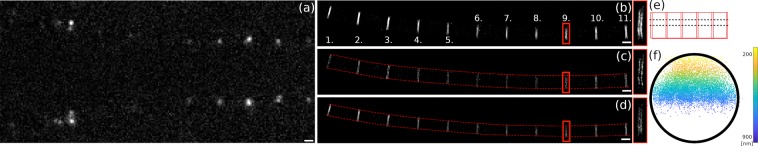
Implementation of biplane 3D imaging in mmSTORM. The primary and secondary PSFs show circular shapes in a single image frame. (**a**) 2D super-resolved (**b**) and cross-sectional (**c**,**d**) dSTORM images show the two discs separately. The side-view of 4th H-zone (**f**) reveals the disk shape with a radius of 0.8 μm. The scale bar is 1 micron.

The biplane 3D STORM modality can be implemented via introducing a slight defocus between the primary and the secondary images. Figure 5 presents the biplane modality in a similar way as astigmatism was discussed in Fig. 4 using an isolated myofibril (with labelled Tmod) stuck parallel to the coverslip. The optimum value of defocus depends on both the sample and the required axial resolution and can be easily set by axial shift of the Porro prism and the secondary objective. During these experiments the axial separation of S and P images was 350 nm. The captured PSFs either in the P and S channels (upper and lower sections in Fig. 5(a)) have cylindrical symmetry and both their relative intensity and width depend on the axial position of the dye molecule. Figure 5(b) shows the traditional 2D super-resolved image based on the evaluation of the primary PSFs only. Separation of the two labeled discs in an H-zone was measured to be approximately 125 nm. The axial position of a fluorescent dye molecule can be determined by comparing its primary and secondary PSFs. Figure 5(c,d) depict two cross-sectional images at planes schematically marked in Fig. 5(e). The widths of the myofibril (marked with dashed red lines) depend on the depth of the sections, however they are independent of the lateral position because (in contrast to the astigmatic measurement) the myofibril was parallel to the coverslip. The axial separation of the two cross-sectional images was 183 nm Fig. 5(e). The radius of the fitted circle to the measured 3D data was 0.8 μm in good agreement with the astigmatic 3D results (Fig. 5(f)).

The STORM images were generated using localizations with precision of better than 50 nm. The mean and median values of these accepted localizations were 19.93 nm and 15.6 nm, respectively. The axial localization precision was calibrated using fluorescence bead, and given as the standard deviation of the calculated Z positions. Based on the evaluation of measured data of 22 beads the axial localization precision and the axial range were found to be 47 nm and 700 nm, respectively.

### Multicolor dSTORM modality

**Figure 6:**
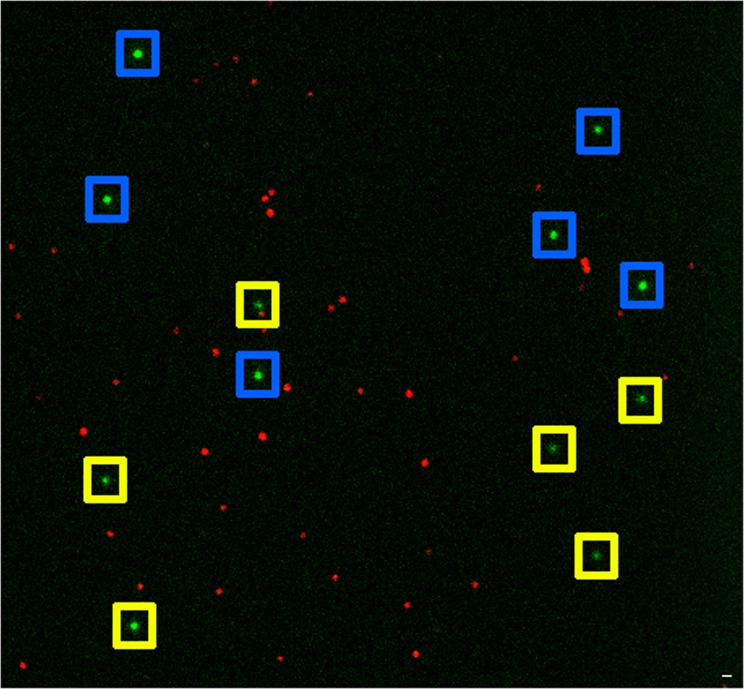
Two-color imaging of fluorescent beads. Beads that can be excited at 561 nm (green dots) have secondary images, while others excited at 647 nm are singlets (red dots). Primary and secondary images of green beads are framed in blue and yellow. The scale bar is 1 micron.

The generated P and S images can also be spectrally separated by inserting an emission filter into the reference arm (Fig. 1(b,iii)). Figure 6 shows an artificially colored image of the mixture of two different kinds of fluorescence beads (green beads: Applied Microspheres BV 30200 FR-CM-M, LOT: 171122 and red beads: Life Technologies T7279, LOT: 1929718) fixed on the coverslip. The beads were simultaneously excited at 647 nm and 561 nm wavelengths, and an additional emission filter for 561 nm excitation was inserted into the reference arm (Semrock FF01-582/75-25). Since this filter blocks the emission of the red dyes, only the beads excited at 561 nm have secondary images. Therefore, the pair finding algorithm practically sorts out the beads based on their emission spectra. In Fig. 6, P and S images of the green beads are framed in blue and yellow, respectively, based on their relative intensity values. During this particular measurement the full FOV (512 × 512 pixels^2^) was excited, which resulted in a spatial overlap of the two images. Such an experiment proves that at low labeling density even the full frame of the detector can be applied and the proposed method does not degrade the FOV. The advantage of this configuration is that the minimum spatial offset between the two images reduces optical aberrations. However, we have to mention that implementation of multi-color dSTORM imaging using fluorescence dye molecules raises several technical challenges. Switching between different dyes simultaneously requires an optimum photochemical environment and it only works for specific dye pairs^51,52^.

## Discussion and Conclusion

A multimodal single molecule localization microscopy technique was developed, optimized and tested. The emitted photons of single fluorescent molecules were collected by a dual objective setup, and primary and secondary mirror images were captured simultaneously on a single detector. Comparative simulation and experimental study proved the feasibility and applicability of the secondary image for SMLM. It was also shown that the overall quality of the primary image was not affected, since the secondary image was generated independently by photons emitted in the direction opposite to the primary objective. (moreover false positive localizations can be eliminated by an optimized pair finding algorithm). Astigmatic and biplane 3D imaging techniques were demonstrated using sarcomere structures, while two-color imaging was tested by means of beads. At low labeling density (approximately half of the normal value applied for dSTORM) the full camera frame can be applied, and the proposed method does not degrade the FOV. Future work will focus on polarization and spectral sensitive detections through the insertion of a polarizer and dispersion prism into the secondary arm. We are also planning to redesign the setup and build a more compact setup based on a knife edge prism that reduces the size of the unusable area between the S and P images.

## Supplementary information

mmSTORM: Multimodal localization based super-resolution microscopy – supplementary materials

## Data Availability

The measured and generated datasets during the current study are available from the corresponding author on reasonable request.
