# Supplementary material for: mmSTORM: Multimodal localization based super-resolution microscopy
Source: Sci Rep. 2019 Jan 28;9:798. doi: 10.1038/s41598-018-37341-9 (PMC6349879; doi:10.1038/s41598-018-37341-9)
Supplement: Supplementary file 1 — mmSTORM: Multimodal localization based super-resolution microscopy – supplementary materials [file 41598_2018_37341_MOESM1_ESM.pdf]

mmSTORM: Multimodal localization based super-resolution microscopy – supplementary materials

Tamás Gajdos[1], Zsófia Cserteg[1], Szilárd Szikora[1,2], Tibor Novák[1], Bálint Barna H. Kovács[1], Gábor Szabó[1,3], József Mihály[1,2], and Miklós Erdélyi[1,]*

[1]Department of Optics and Quantum Electronics, University of Szeged, 6720 Szeged, Dóm tér 9, Hungary
[2]BRC Institute of Genetics, Biological Research Centre HAS, Szeged, Hungary
[3]MTA-SZTE Research Group on Photoacoustic Spectroscopy, Szeged, Hungary
[*]Corresponding author: erdelyim@physx.u-szeged.hu

ABSTRACT

This document provides supplementary information to "*mmSTORM: Multimodal localization based super-resolution microscopy*". In the first section (Optical setup) an expanded technical description of the optical arrangement is given helping potential users to reproduce the experiments. In the second section the applied OSLO lens is presented and the axial distributions of primary and secondary PSFs are compared. In the third section statistical comparison of the primary and secondary images of actin filaments is given based on histograms of localization precision, standard deviation and intensity of measured PSFs. The TestSTORM simulation, provides a better understanding of the captured super-resolved images of myofibrils. In the last section we give a detailed protocol of sample preparation.

Optical Setup

The applied microscope system is based on a Nikon Eclipse Ti-E frame. A custom-made relay system (Figure S1) was built over the microscope using a Nikon CFI Plan Apo Lambda objective (NA 1.4, 60x magnification, oil immersion), a 2” dielectric mirror (Thorlabs BB2-E02), a lens with a focal length of 250 mm (Thorlabs AC254-250-A-ML), and an AR-coated right angle prism (Porro prism: Thorlabs, PS908H-A). A single-axis translation stage (Thorlabs PT1) with a custom built stepper motor actuator was used to align the second objective’s (O2) z-position. The stage was moved by 1/4"-80 threaded fine hex adjuster (Thorlabs F25ST200) inside a barrel adapter (Thorlabs F25SSA1). The other end of the hex adjuster was inside the custom machined 4 cm aluminum tube, which was glued to a flexible shaft coupler. A NEMA17 size bipolar stepper motor was connected to this coupler, which was driven by an Arduino Uno R3 and a Motor Shield v1. A custom Arduino code and a LabView user interface was developed, which can be downloaded from GitHub[1]. During the measurements HILO and EPI-fluorescent illuminations were applied at excitation wavelengths of 647 nm (2RU-VFL-P-300-647-B1, 300 mW, MPB Communications Ltd.) and 561 nm (Cobolt Jive, 300 mW). For reactivation a 405 nm laser diode (Nichia NDHV310APC) was used. In case of dSTORM measurements, the applied excitation intensity was ~4 kW/cm2. In the primary channel a Nikon CFI Apochromat TIRF objective (NA 1.49, 100× magnification, oil immersion) was used for imaging. A filter set from Semrock was used in the microscope (Di03-R405/488/561/635-t1-25x36BrightLine® quad-edge quad-edge super-resolution / TIRF dichroic beamsplitter and FF01-446/523/600/677-25 BrightLine® quad-band bandpass filter) for spectral separation of excitation and emission lights. In single color (647 nm excitation) imaging an additional filter was placed in the detection path for background reduction (BLP01-647R-25 647 nm EdgeBasic™ long-pass edge filter). An Andor iXon3 DU897 EMCCD camera was used for image acquisition with a pixel size of 16 µm. The dSTORM measurements were carried out with the following acquisition parameters: 20 or 30 ms exposure time, EM gain of 100, temperature of -75 °C.

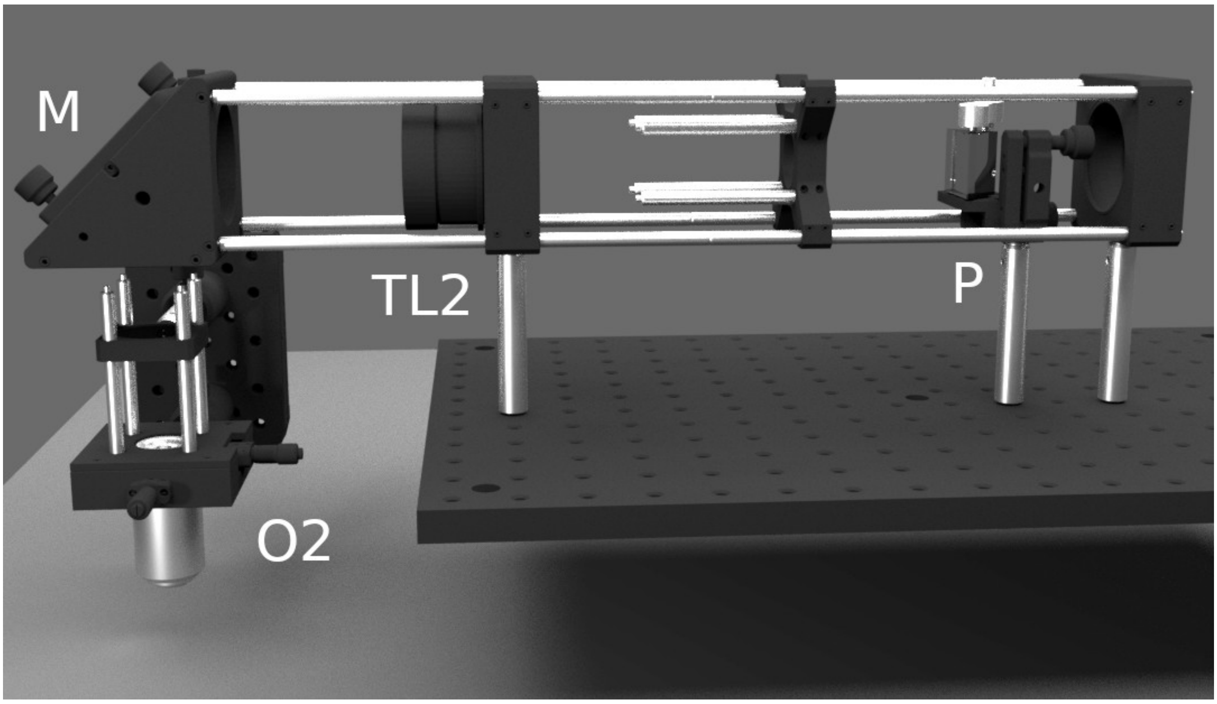

Figure S1. *Simulated 3D view of the proposed multimodal setup inserted above the sample (O2: objective; M: 2 inch mirror; L2: achromatic lens; P: AR coated Porro-prism)*

The sample was mounted between two coverslips (typically onto the upper one), where the thickness of the sample (including the buffer) was kept below 25 microns. The coverslip "sandwich" was mounted on a custom made 2 mm thick metal sample holder with a 2 cm diameter hole in the middle. The z position was measured from the surface of the upper coverslip.

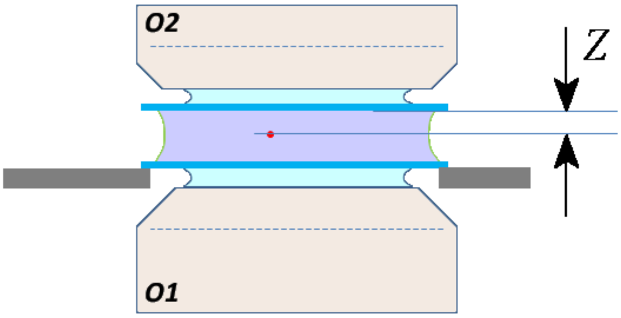

Figure S2*. objective-oil-coverslip-sample-coverslip-oil-objective sandwich with the sample holder and definition of axial z position of the fluorescent point-like source*

We used this geometry on purpose: the primary objective was a Nikon TIRF objective with a cover glass correction collar (0.11-0.19) so we could compensate the 25 micron thin PBS layer. In contrast, the secondary objective was a Nikon CFI Plan Apo Lambda objective corrected for standard 0.17mm thick coverglass. It is worth noting here that the secondary objective was a PLAN objective. Therefore, we believe that such a configuration can be set for imaging the sample fixed to the upper coverslip with minimum optical aberrations.

Implementation and image alignment protocol

I. Align EPI illumination mode so that the excitation beam marks the optical axis.
II. Install the mirror (M) and set its position/orientation so that the beam runs through the center of all the holders in the cage system.
III. Install the secondary tube lens (L2) 25 cm far from the back focal plane of the secondary objective and center it using the transmitted and reflected beams.
IV. Install the right angle prism (P) 25 cm from the tube lens using a prism mount. Set its lateral position and orientation so that a reflected beam with identical size as the incoming one appears at the plane of the sample.
V. Remove the primary objective and install the secondary one into the XYZ translation stage. Align the stage to center the back reflected beam. Reinstall the primary objective.
VI. Insert a sample and adjust the z position of the primary objective to image the upper coverslip. Turn off the auto scale function (in Nikon NIS software) and adjust the dynamic range to make the laser spot on the sample visible.
VII. Adjust the z position of the secondary objective until the secondary image appears on the camera. Set the position of the secondary objective (using the smallest step size) so that the edge of the prism can be seen with the highest contrast. In this case the primary and secondary object planes are identical. Using beads as a test sample refine the z position of the secondary objective.
VIII. Optional: The Nikon PFS can lock onto the upper coverslip, and keep the primary objective (O1) in focus. Using a piezo stage the secondary object plane can be further adjusted by moving the sample, while keeping the primary one fixed.
IX. If necessary, adjust the lateral position of O2 to center the prism's edge.
X. Install an aperture into the conjugate plane of the object in the excitation path to block half of the excitation beam.
XI. To remove the reflected excitation beam TIRF or HILO illumination can be set, or optionally an emission filter can be installed into the secondary imaging path.

Stability considerations

The proposed optical configuration was implemented using standard optical components (mainly purchased from Thorlabs). We preferred to apply a cage system because of its long-term stability. During the measurements special care was taken to minimize drift: the whole system was covered and shielded, the system was turned on several hours before the measurement so that the system was in thermal equilibrium etc.. Despite these efforts, the stability of the secondary arm was sometimes lower than the primary one, probably because of the instability of the optical stages and the homemade mount of the sample. Therefore, we are working on replacing all the optical mounts in the secondary arm and design a robust and easily implementable tool.

Computer simulation of the optical setup

Unfortunately, exact comparative simulation and experimental study was difficult, because the manufacturer did not provide information on the applied objectives. For the simulations we tried to select objective models with parameters (manufacturer, numerical aperture, magnification, working distance) as close to the real values as possible. We know the limitations of the applied models (without AR coating the absolute value of the calculated Strehl-ratio is overestimated, missing coverglass correction collar introduces a different value of spherical aberration etc.) and therefore we used the simulation results with utmost care. Although during the experiments the primary objective was corrected 0.17mm thick glass coverslip and approx. 25 micron thin PBS layer, in the simulations both the primary and secondary objectives were corrected for standard 0.17mm thick coverglass. Despite the clear discrepancy between the simulation and experimental conditions we believe the simulations provide important information on the proposed system because:

I. We know that the sample can introduce serious optical (mainly spherical) aberrations. However, dynamic alignment of the coverslip correction collar during the measurement is only possible in the most up-to-date microscope frames. Therefore, the end users must set the collar before the sample is placed into the holder. Since the thickness of the sample is usually unknown or users intend to image different sections of the sample, sometimes the users keep the standard 0.17 mm correction setup. Therefore, simulation results under this “not optimized” condition is still interesting in practice.
II. The spherical aberration introduces a cylindrical symmetry distortion that reduces the localization precision, but does not affect the central position of the fitted Gaussian distribution. Therefore, simulation results focus on relative and not absolute merit values of PSF.
III. Spherical aberration reduces the image quality of the secondary image. Both the simulations (see image S4) and the experiments (see Figure 2 c and d) show this degradation. However, the primary PSF shows minimal asymmetry (see image S4) despite the fact the objective is corrected for 0.17mm thick coverglass. We believe the reason behind this almost perfect PSF is the optimized oil-coverglass-PBS thickness. In other words, the reduced oil thickness can partially compensate the PBS (sample) thickness.

The optical system was modeled by means of OSLO (Optics Software for Layout and Optimization). The applied microscope objectives[2,3] were implemented into the code (Figure S3) and both the lateral and axial distribution of the PSF was studied as a function of the axial position (and defocus) of the point-like source (Figure S4). The knowledge of PSF distortion as a function of the axial position is extremely important in 3D imaging since it provides information on both the maximum axial range of 3D imaging and its dependence on depth.

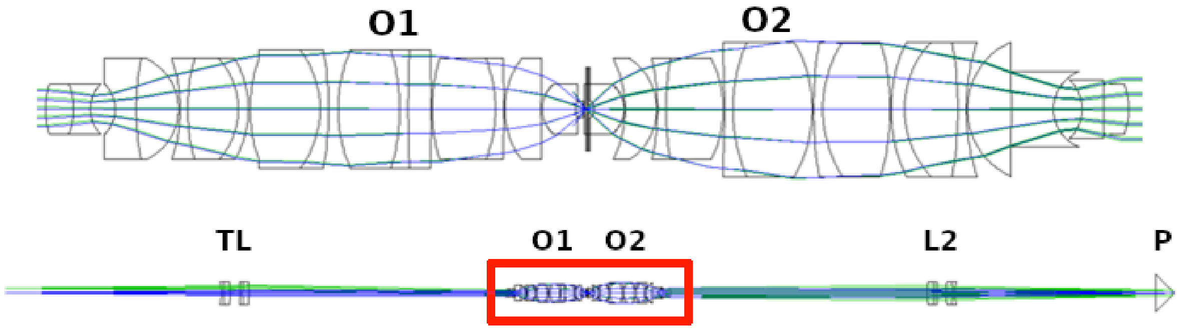

Figure S3. *2D layout of the system generated by OSLO. The sample inserted between the two microscope objectives can be imaged either directly via the left (primary) objective or indirectly by generating a secondary image first via the right (secondary) objective*

Figure S4 shows the impact of the thin, 25 micron PBS layer on both the primary and secondary PSFs when the actual object plane is 0, 1, 2 and 4 microns away from the upper coverslip. The computer simulation reveals that the secondary image suffers from spherical aberration and the Strehl ratio is typically reduced by 50%. Despite this image degradation the secondary image can be used for multimodal dSTORM imaging as detailed in the main text.

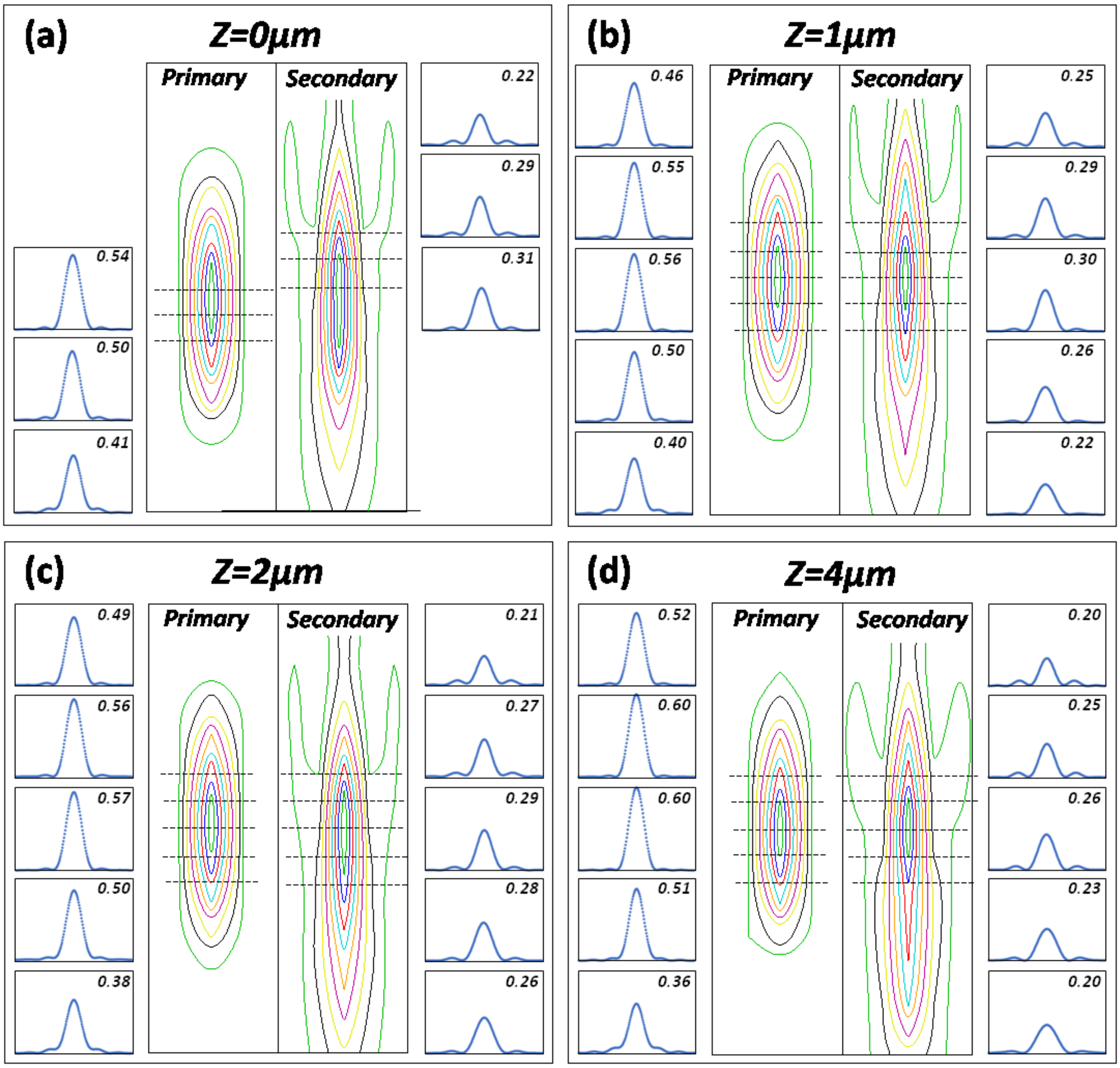

Figure S4. *Comparative simulations for primary and secondary PSFs (axial and lateral dependence) in OSLO. The calculated Strehl ratio values are marked in the insets.*

Comparative evaluation of the primary and secondary localizations

The statistical evaluation of primary (a-c) and secondary (d-f) images is presented in Figure S5 using the histograms generated by the built-in module of rainSTORM[4]. This comparison shows decreased brightness (a,d), broadened FWHM (b,e) and reduced localization precision (c,f) in the secondary channel. Despite this image degradation, PSF captured in the secondary channel could be applied to mmSTORM imaging such as 3D, spectral and polarization sensitive detection.

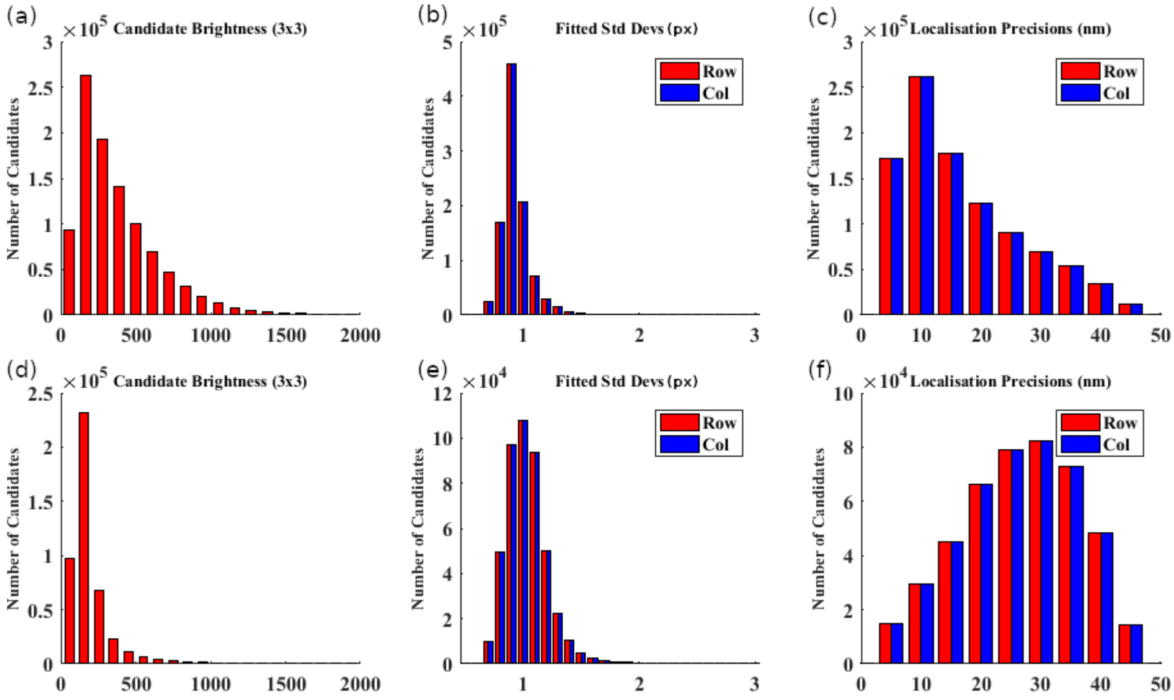

Figure S5. *Histograms of measured primary and secondary images of F-actin sample. The brightness was given in camera counts (conversion factor: 1.357 photon/count). The pixel size was 160 nm.*

Optimization of the introduced astigmatism based on paraxial matrix optics method and experimental calibration

Paraxial matrix optics based calculation was used to optimize both the focal length and the insertion point of the cylindrical lens. The system matrix of the simplified optical arrangement (Figure S6) can be given as the product of the matrixes of the individual optical components[5].

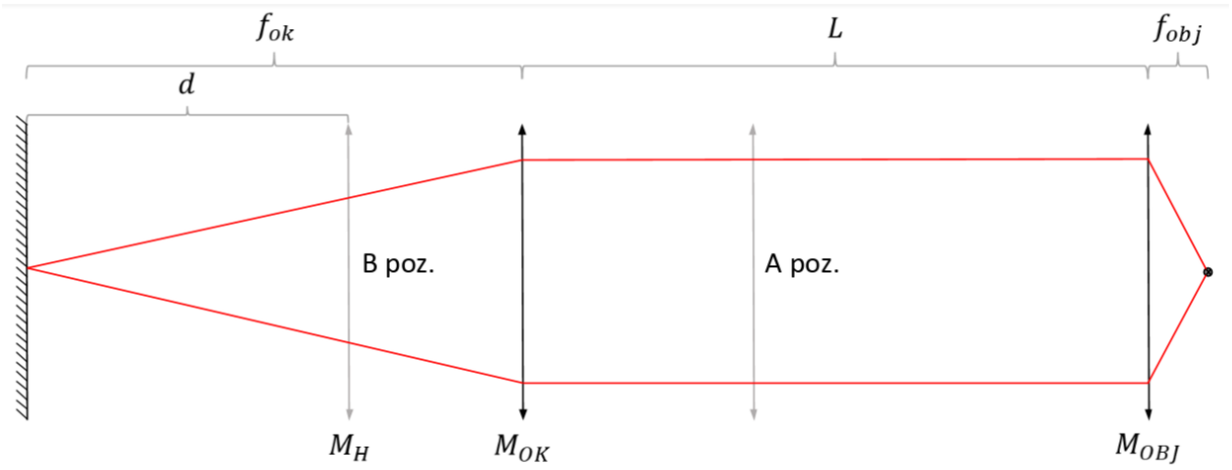

Figure S6. *Schematic view of the simplified optical setup used for paraxial matrix optics based evaluation of astigmatism.*

Two arrangements were evaluated depending on the insertion point of the cylindrical lens. In configuration A the cylindrical lens was placed between the secondary objective (O2) and the tube lens (L2), while in configuration B the lens was behind the tube lens. The position of the inserted cylindrical lens was measured from the mirror, which functioned as the applied Porro prism applied in the measurements. In both cases rays travelled through the optical components twice (forward and back) and the system matrixes could be given as:

$$M_{System}^{(A)} = T_k \cdot M_{O2} \cdot T_{(L+f_{L2}-d)} \cdot M_{Cy} \cdot T_{(d-f_{L2})} \cdot M_{L2} \cdot T_{(2\cdot f_{L2})} \cdot M_{L2} \cdot T_{(f_{L2}-d)} \cdot M_{Cy} \cdot T_{(L+f_{L2}-d)} \cdot M_{O2} \cdot T_{(f_{O2})}$$

and

$$M_{System}^{(B)} = T_k \cdot M_{O2} \cdot T_{(L)} \cdot M_{L2} \cdot T_{(f_{L2}-d)} \cdot M_{Cy} \cdot T_{(2\cdot d)} \cdot M_{Cy} \cdot T_{(f_{L2}-d)} \cdot M_{L2} \cdot T_{(L)} \cdot M_{O2} \cdot T_{(f_{O2})},$$

where M_{O2}, M_{L2}, M_{Cy} and T_l are the ray transfer matrixes of O2 objective, L2 tube lens, the cylindrical lens and the translation of distance l.

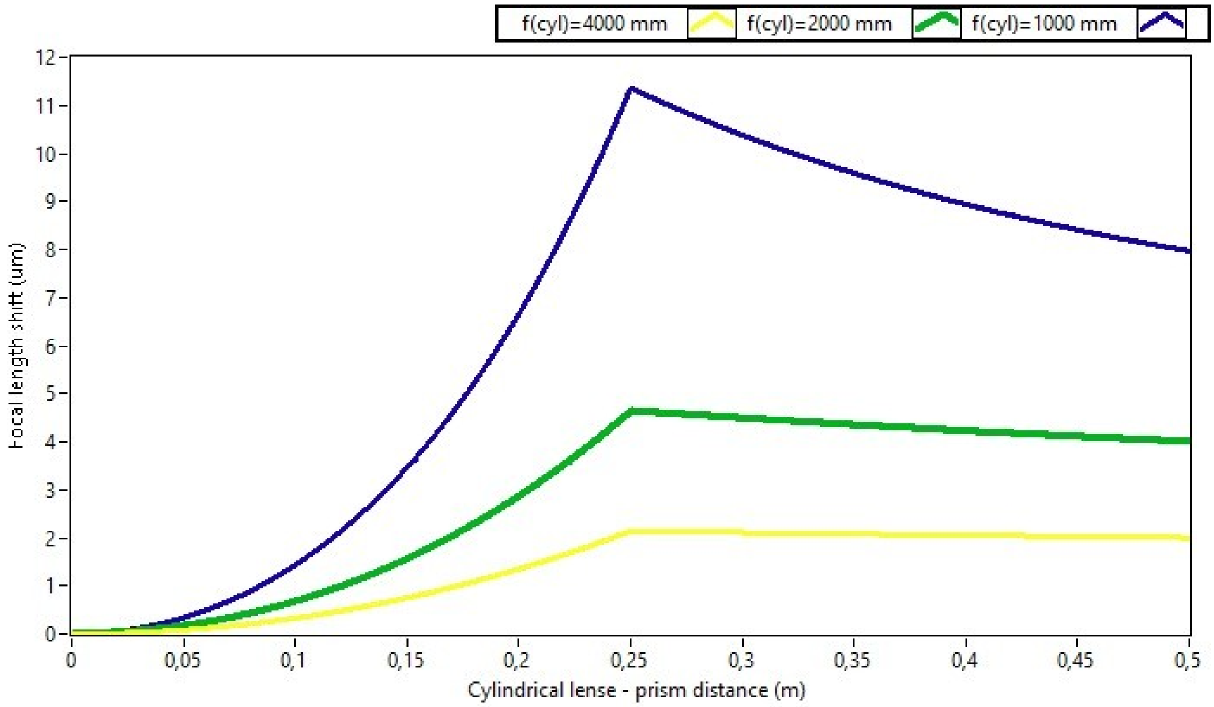

Figure S7. *Introduced focus separation a function of the focal length and insertion point of the cylindrical lens.*

The focal lengths with (k) and without (fO2) the insertion of the cylindrical lens were calculated and their difference was used to quantify the introduced astigmatism. The positions of the foci were determined by using the theorem that under stigmatic imaging the matrix element M12 of the entire system has to be zero. Figure S7 shows the separation of these sagittal and tangential foci a function of the axial position of the cylindrical lenses with focal lengths of 1000, 2000 and 4000 mm. Between the objective and the tube lens the divergence of the beam is small therefore the value of astigmatism mainly depends on the focal length of the inserted cylindrical lens and shows moderate dependence on the focal length. In contrast, behind the tube lens the effective NA of the cylindrical lens (radius of illuminated disk on the lens/focal length) strongly depends on its position therefore astigmatism can be relatively easily tuned by the axial position of the lens.

Using fluorescent beads multiple experimental calibration curves were captured with different cylindrical lens insertion points. Figure S8 (a) shows the evaluated measurement data for the 95 mm lens distance, and the calculated ellipticity (b). For each lens position the ellipticity slope was calculated (c).

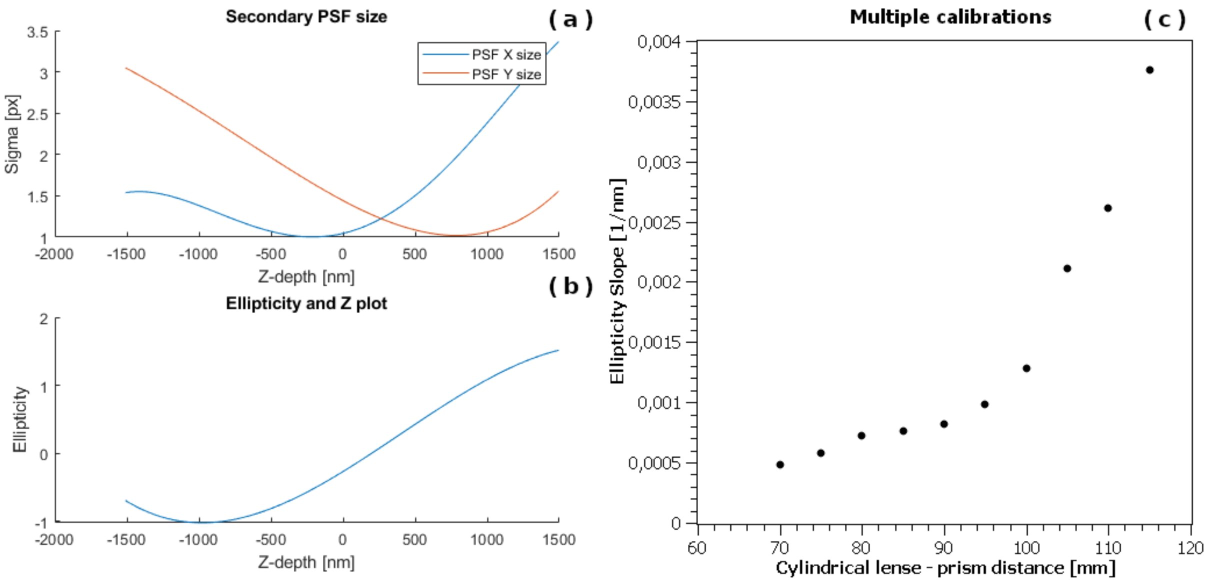

Figure S8. *Experimental calibration curve recorded with fluorescent beads, when the cylindrical lens was placed 95 mm from the prism (a, b). The calculated ellipticity slopes are shown as a function of the insertion point of the cylindrical lens in the secondary arm (c).*

3D astigmatic imaging of microtubules

Schneider 2 cells were imaged using the mmSTORM optical setup with a cylindrical lens (Comar 4000 Y 25) inserted into the secondary arm. Both objectives were adjusted to image the same plane near the upper coverslip, and the perfect focus system (Nikon PFS) was enabled. During the dSTORM measurement 20,000 frames were captured with the exposure time of 30 ms. The image stack was evaluated with rainSTORM using the 'Least-Squares Astigmatic Gaussian 2D const Bg' algorithm, and the generated event list was filtered for localization precision better than 30nm. The pair matching was done using the algorithm described and presented in the Pair finding algorithm section of Supplement.

Figure S9 (a) was reconstructed with the Simple Histogram method, using 26.66 nm as super-pixel size (scale factor: 6). Figure S9 (b) was reconstructed using the X-Y coordinates from the primary localizations, and the Z coordinates from the secondary localizations. The final image was created using the mean Z values for each pixel.

The microtubules near the coverslip (in blue) can be imaged with good X-Y precision. The microtubules closer to the nucleus (in yellow) are out of focus of the primary objective, and the X-Y precision degrades.

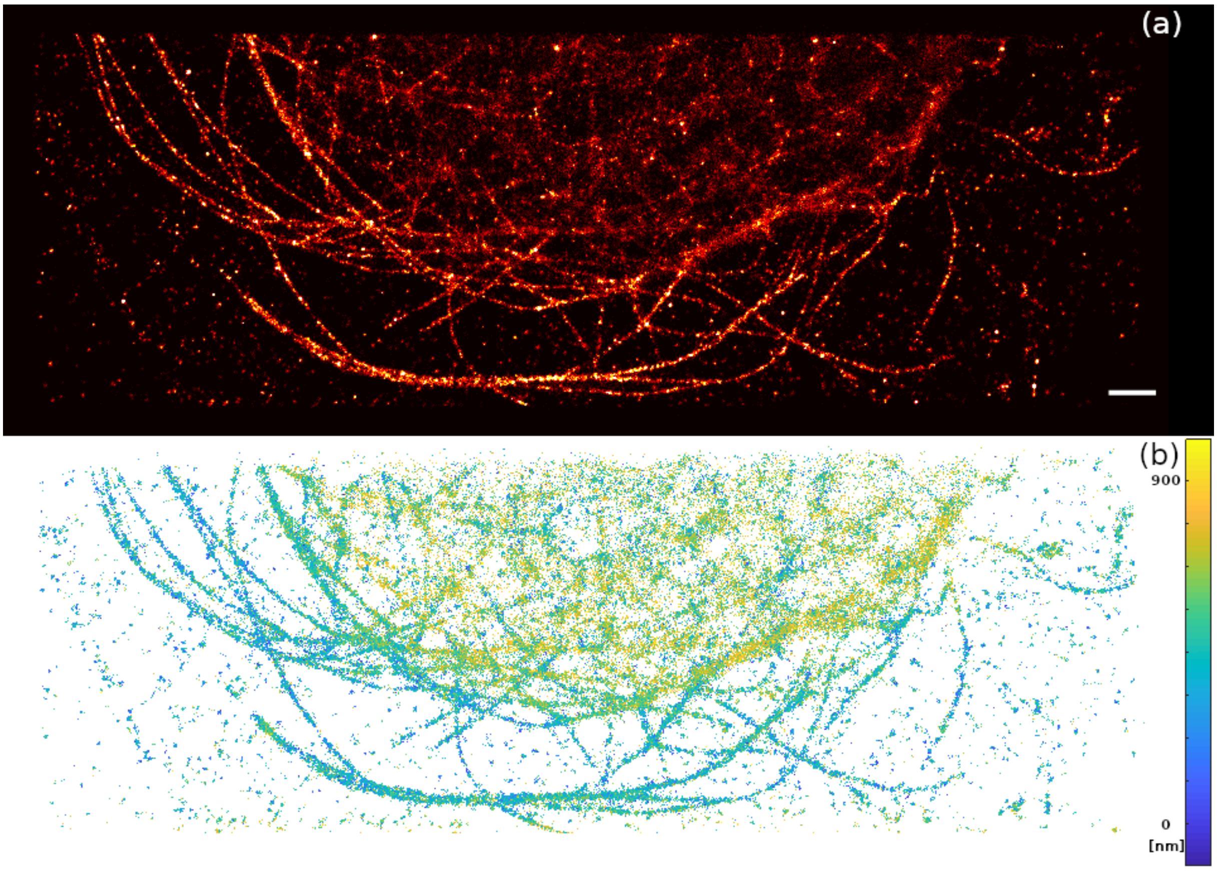

Figure S9. *2D and 3D reconstruction of the microtubule network of an Schneider 2 cell. The scale bar is 1 micron.*

TestSTORM simulation of sarcomere structure

dSTORM imaging of tilted myofibrils was also studied using simulated image stacks generated by a TestSTORM v2.0.3 code[6] with the following parameters:

- Distance of discs: 130 nm
- Radius of discs: 600 nm
- Distance of zones: 1200 nm
- Number of zones in a single myofibril: 7
- Number of myofibrils: 6
- Tilt of myofibrils: 0°, 2°, 4°, 6°, 8° and 10°
- Image size: 64x64 pixels
- Number of frames: 40000
- Labeling density: 40
- PSF mode: Gaussian

The visibility of the double disc structures strongly depends on the orientation of the sample and the applied filtering conditions in rainSTORM code.

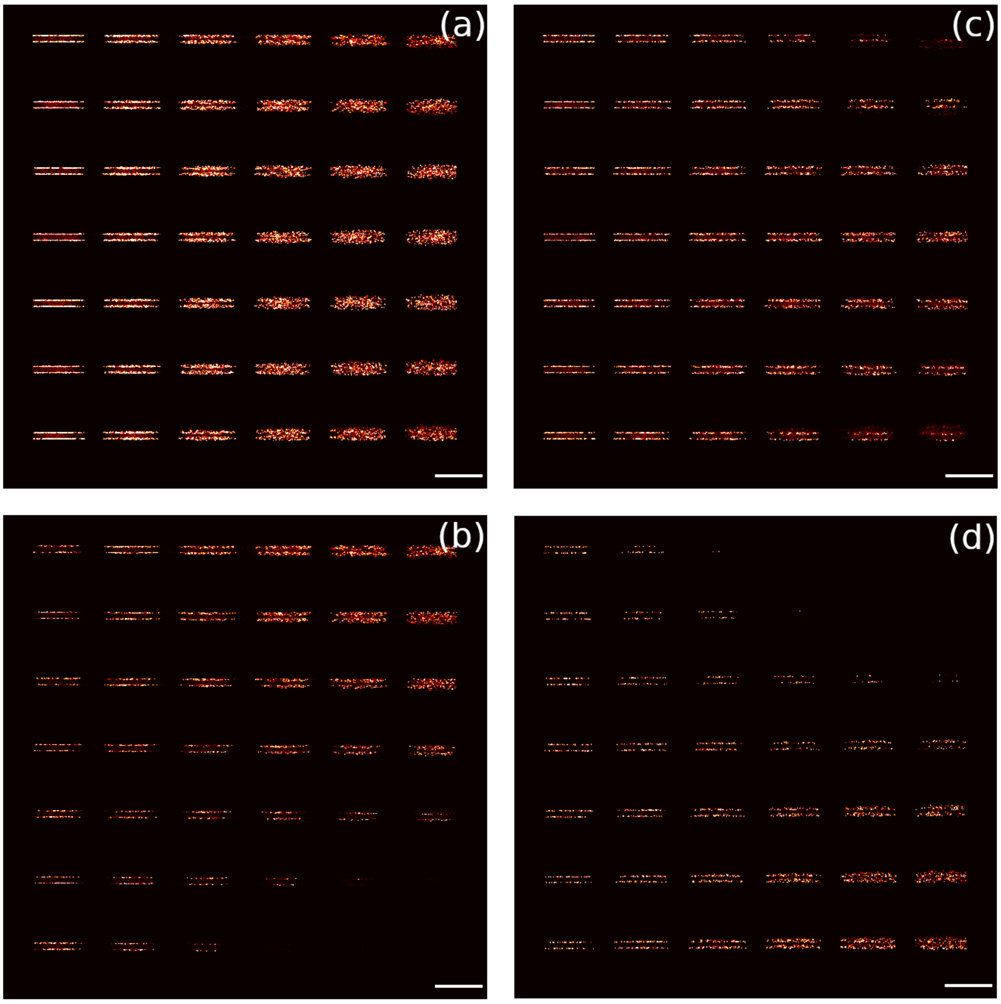

Figure S10. *TestSTORM simulation for tilted sarcomeric structure unfiltered (a) and filtered for ellipticity (b): (-inf, -0.6), (c): (-0.6, 0.6) and (d): (0.6, inf). The The scale bar is 1 micron.*

Tissue preparation and immunostaining

Drosophila flight muscle tissue sample

For SMLM microscopy individual myofibrils were isolated from the Indirect Flight Muscles of anesthetized adult (~24 hours after eclosion) Drosophila as described previously[7], with minor modifications.

In brief, bisected hemi-thoraces were incubated in relaxing solution (100mM NaCl; 20 mM NaPi pH, 7.0; 5 mM MgCl2; 5 mM EGTA; 5 mM ATP) supplemented with 50% glycerol for 2 hours at 4 °C. Afterwards the dorsal longitudinal muscles were isolated from the hemi-thoraces and gently dissociated by pipetting them in an Eppendorf tube in the presence of 0.5% Triton X-100. Dissociated myofibrils were centrifuged at 10000 rpm for 2 minutes. Myofibrils were washed and centrifuged two more times in relaxing solution. Myofibrils were re-suspended in relaxing solution and 20 µl of the sample was dropped on a glass coverslip and fixed with 4% paraformaldehyde (Alfa Aesar) in relaxing solution for 15 min. After being washed three times in relaxing solution the samples were blocked in blocking solution (5% goat serum (Sigma), 0.1% Triton X-100 in relaxing solution) for 30 min in a humidity chamber. Tropomodulin antibody (rat, 1:200) was applied overnight at 4 °C in blocking solution. Following the washes, Alexa Fluor 647 (1:600, life technologies, goat anti-rat IgG) secondary antibody was applied for two hours at room temperature. The samples were embedded in GLOX-MEA imaging buffer before super-resolution dSTORM measurements.

Flies (w1118) were raised at 25 °C according to standard procedures.

Schneider 2 cell cultures and immunohistochemistry

Schneider 2 (S2) cells were grown in Schneider’s media supplemented with 10% heat inactivated FBS and penicillin-streptomycin solution. For immunostaining, 2x106 S2 cells were seeded in 6-well plates and fixed after an hour. Samples were initially fixed and extracted for 1 min using a solution of 0.3% (v/v) glutaraldehyde and 0.25% (v/v) Triton X-100 in cytoskeleton buffer (CB, 10 mM PIPES, pH 7, 150 mM NaCl, 5 mM EGTA, 5 mM glucose and 5 mM MgCl2), and then post-fixed for 15 min in 2% (v/v) glutaraldehyde in CB (Xu et al., 2012). The samples were briefly washed in CB, then permeabilized and blocked in blocking buffer (5% w/v goat serum, 0.2% v/v Triton X-100 in CB) for 30 min. Microtubules were labelled using an anti-α-tubulin (1:1000; DM1A; Sigma) primary antibody for 2 hours at RT. For secondary antibody, we used the appropriate Alexa-647 conjugate for an hour at RT.

Pair finding algorithm

In this section X and Y coordinates on the single image frames are referred to as non-mirrored and mirrored coordinates. Assuming a perfect alignment (the edge of the prism is centered, primary and secondary images are not rotated etc.) the sum and difference of the mirrored (X, X') and non-mirrored coordinates (Y, Y') of a pair is constant (and equals to the frame size) and zero, respectively. However, optical aberrations distort the captured images and additional processing steps are necessary to find the real pairs. Shortly, the most critical steps of the applied pair finding algorithms are the following:

I. Rough estimation for the sum of mirrored coordinates is given using the minimum and maximum mirrored coordinates of localizations on a single frame. The initial value of difference of the non-mirrored coordinates was zero.
II. Calculation of the sum and difference non-mirrored and mirrored coordinates of all the possible localizations on 1000 frames.
III. Histogram generation and refine the sum and difference values.
IV. Calculation of the theoretical position of the pair of a localisation.
V. Judge and select the nearest localization using the calculated pairing cost.

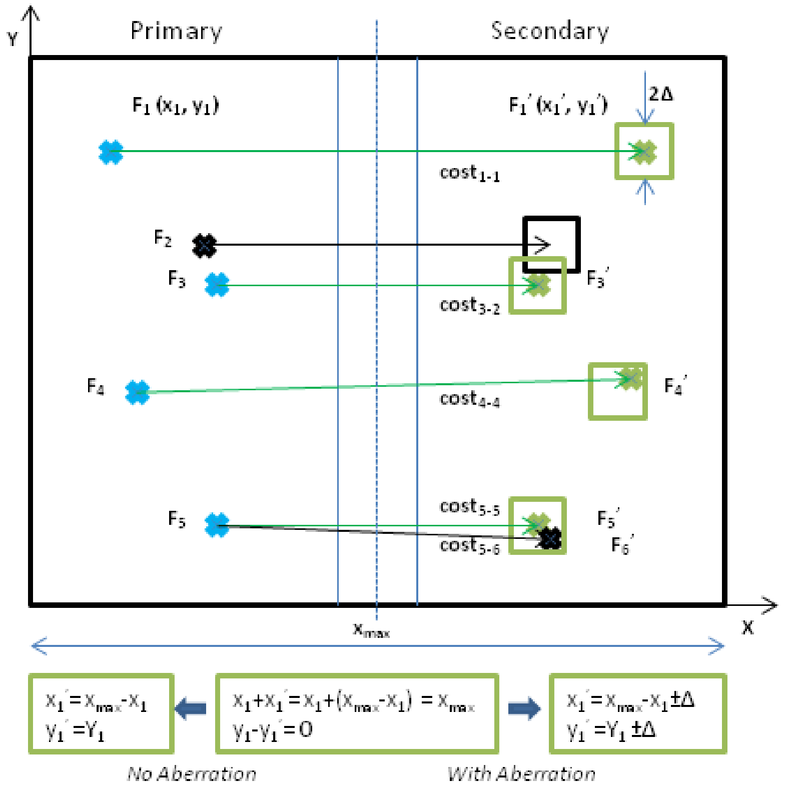

Figure S11*. Judge and selection examples: F_1-F_1' is a perfect match; F_2 has no pair in the permit window, thus it is discarded; F_3-F_3' is also a perfect match; F_4-F_4' pair is accepted as the secondary localization is inside the permit window; F_5-F_5' is accepted and F_5-F_6' is discarded as the latter has a higher pairing cost.*

```
%%    Summary:    Pairing script built for pol-dSTORM, modified to work with mmSTORM
%     Version:    0.4 - disabled histogram plot, instead returned as a variable
%     Note:      Added the ability to externally iterate.
%     Package:    rainSTORM v3.2+     (Using postprocessing)
%     Developer:    gajdost (based on the pairing code from nemethj)
%     Company:    University of Szeged, Advanced Optical Imaging Group
%     Comment:  Finds the pairs based on difference in Y and sum in X.
%     License:  GPLv2
function [paired, distance, hist] = pairing_mm( SupResParams, modalityParams )
paired = [];   % Initial clear
% Load here the localization parameters from the eventlist.
data.x_coords = [ SupResParams.x_coord]'; % in px
data.y_coords = [ SupResParams.y_coord]'; % in px
data.frameIndices = [ SupResParams.frame_idx]';
data.id = [ 1:length( SupResParams ) ]';    % Localization id.

% Get the parameters from the struct
maxXDiff = modalityParams.maxXDiff; % Maximum allowed difference in X
maxYDiff = modalityParams.maxYDiff; % Maximum allowed difference in Y
if isfield(modalityParams,'distance')   % If there is a previous guess, use that
        edge = modalityParams.distance; display(['Previous guess: ' num2str(edge)]);
else    % In case of first run, use the edges, not the best guess, but a good one.
        edge = round(min([SupResParams(:).x_coord]) + max([SupResParams(:).x_coord]));
end
maxFrames = max( data.frameIndices );

mySum  = @(v1, v2) bsxfun(@plus, v1', v2); % My framesize: x_1 + x_2 = d
myDist = @(v1, v2) bsxfun(@minus, v1', v2);
% Craft histogram(TM) initialization.
hist = zeros( 1+ceil(edge+maxXDiff) , 1 );
% Calculate the sum of the coordinates = frame size.
if ( maxFrames >=2000)
        disp('Using [1000 1999] frames for initial guess');
        maxFrame = 1000; deltaFrame = 999;
else
        disp(['Using ', num2str(maxFrames), ' frames for initial guess']);
        maxFrame = maxFrames; deltaFrame = 0;
```

```
end

% We do an actual guess!
for i = (1+deltaFrame):(maxFrame+deltaFrame)

       X = data.x_coords( data.frameIndices == i );
       Y = data.y_coords( data.frameIndices == i );
       sX = mySum( X, X );
       dY = myDist( Y, Y );

       sums = sX( abs(dY) <= maxYDiff & sX>= edge-maxXDiff );
       % The idea is that they can only differ slightly in Y, and their
       % sum in X is approx. the size of the frame.
       % Now we try to get the maximum out of our craft histogram.
       for j = 1:length( sums )
       S = round(sums(j));
       if (S < (edge+maxXDiff)), hist( S ) = 1 + hist( S ); end
       end
end

hist = conv( hist, fspecial('gaussian',[3 1], 1.0), 'same');
% We get the most probable distance and we do the actual pairing with it.
distance = find( hist == max(hist ) ); %    figure; plot( hist );
% Actual pairing
for i = 1:maxFrames
       if (mod(i,1000) == 0), disp(['Frame: ', num2str(i), '/', num2str(maxFrames)]); end
       X = data.x_coords( data.frameIndices == i );
       Y = data.y_coords( data.frameIndices == i );
       indices = data.id( data.frameIndices == i );
       sX = mySum( X, X );
       dX = myDist( X, X );
       dY = myDist( Y, Y );
       % We use this neat cost function, where the deviations are squared.
       % The penalty gets really high if the deviation is large
       costMat = abs((abs(sX) - distance).^2 + dY.^2);
       % So, if the difference in Y is larger than maxYDiff (def:2) OR
       % The points are closer than 20px (the prism edge) OR
       % The the coordinate sum and distance difference is larger than
       % maxXDiff THEN we don't want the pairing to occur.
       costMat( abs(dY) > maxYDiff | dX<=20 | abs(sX-distance) > maxXDiff ) = inf;
       % Let's do the assignment, based on greedy pairing. From the list
       % the lowest cost pairing will be done.
       assign = greedyPairs( costMat, modalityParams.maxCost );

       for j = 1:length( assign )
       if assign(j)>0, paired(end+1,:) = [ indices(j) indices(assign(j)) ]; end
       end
end
end

function assignment = greedyPairs( costMat, maxCost )
       assignment = zeros( 1, size(costMat,1) );
       if numel( costMat ) <= 0, return; end
       while true
       [x, y] = find( costMat == min(costMat(:)), 1, 'first' );
       if costMat(x,y) == inf, break; end
       if costMat(x,y) >= maxCost, break; end
       costMat(x,:) = inf;
       costMat(:,y) = inf;
       assignment(x) = y;
       end
end
```

References

1. https://github.com/gajdipajti/arduino_stepper_control

2. Mandai, M. & Yamaguchi, K. Immersion microscope objective lens, US patent US 7,046,451 B2, (2004).

3. Yamaguchi, K. Immersion microscope objective lens, US patent US 6,519,092 B2, (2000).

4. Erdélyi, M. & Gajdos, T. rainSTORM User Manual http://titan.physx.u-szeged.hu/~adoptim/?page_id=582

5. A. Gerrard, J. M. Burch: Introduction to matrix methods in optics, Dover Publications, Inc. New York 1994.

6. Novák, T., Gajdos, T., Sinkó, J., Szabó, G. & Erdélyi, M. TestSTORM: Versatile simulator software for multimodal super-resolution localization fluorescence microscopy *Scietific Reports* 7, **951** (2017).

7. Burkart, C., Qiu, F., Brendel, S. Benes, V., Hååg, P. Labeit, S. Leonard, K. & Bullard, B. Modular proteins from the Drosophila sallimus (sls) gene and their expression in muscles with different extensibility *J. Mol. Biol.* **367**, 953-968 (2007).
